# Bis(4-amino­benzene­sulfonamide-κ*N* ^4^)di­chlorido­zinc

**DOI:** 10.1107/S160053681303417X

**Published:** 2013-12-24

**Authors:** Sabrina Benmebarek, Mhamed Boudraa, Sofiane Bouacida, Hocine Merazig, George Dénès

**Affiliations:** a Unité de Recherche de Chimie de l’Environnement et Moléculaire Structurale, CHEMS, Université Constantine 1, 25000, Algeria; b Département Sciences de la Matière, Faculté des Sciences Exactes et Sciences de la Nature et de la Vie, Université Oum El Bouaghi, Algeria; c Laboratory of Solid State Chemistry and Mössbauer Spectroscopy, Laboratories for Inorganic Materials, Department of Chemistry and Biochemistry, Concordia University, Montreal, Quebec, H3G 1M8, Canada

## Abstract

In the title compound, [ZnCl_2_(C_6_H_8_N_2_O_2_S)_2_], the Zn^II^ ion lies on a twofold rotation axis and has a slightly distorted tetra­hedral coordination geometry, involving two Cl atoms and two N atoms from the amino groups attached directly to the benzene rings [Zn—Cl = 2.2288 (16) Å and Zn—N = 2.060 (5) Å]. The dihedral angle between the benzene rings is 67.1 (3)°. The crystal packing can be describe as layers in a zigzag arrangement parallel to (001). The amine H atoms act as donor atoms and participate in inter­molecular N—H⋯O and N—H⋯Cl hydrogen bonds, forming a three-dimensional network.

## Related literature   

For background to sulfanilamides and their applications, see: Wong & Giandomenico (1999 ▶); Ferrer *et al.* (1990 ▶); Supuran *et al.* (1998 ▶); Medina *et al.* (1999 ▶). For related structures, see: Benmebarek *et al.* (2012 ▶, 2013 ▶).
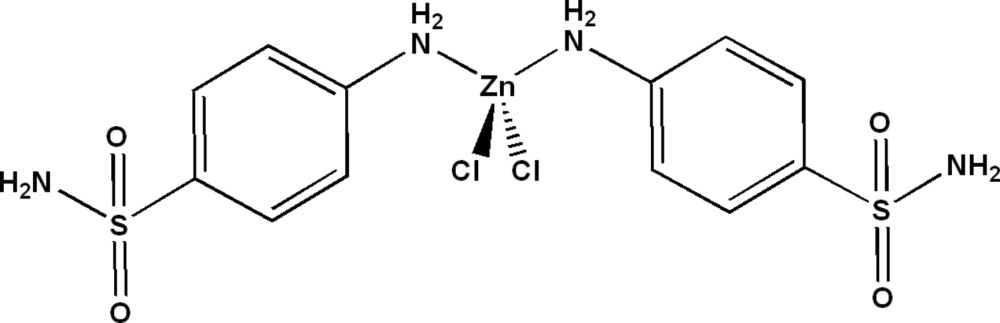


## Experimental   

### 

#### Crystal data  

[ZnCl_2_(C_6_H_8_N_2_O_2_S)_2_]
*M* *_r_* = 480.68
Orthorhombic, 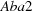

*a* = 7.7957 (15) Å
*b* = 27.916 (6) Å
*c* = 8.2701 (17) Å
*V* = 1799.8 (6) Å^3^
*Z* = 4
Mo *K*α radiation
μ = 1.92 mm^−1^
*T* = 150 K
0.23 × 0.19 × 0.15 mm

#### Data collection  

Bruker APEXII CCD diffractometer
Absorption correction: multi-scan (*SADABS*; Bruker, 2002 ▶) *T*_min_ = 0.639, *T*_max_ = 0.746
6151 measured reflections
2051 independent reflections
1563 reflections with *I* > 2σ(*I*)
*R*_int_ = 0.075

#### Refinement  

*R*[*F* ^2^ > 2σ(*F* ^2^)] = 0.040
*wR*(*F* ^2^) = 0.067
*S* = 1.00
2051 reflections
120 parameters
3 restraints
H atoms treated by a mixture of independent and constrained refinement
Δρ_max_ = 0.38 e Å^−3^
Δρ_min_ = −0.40 e Å^−3^
Absolute structure: Flack parameter determined using 601 quotients [(*I* ^+^)−(*I* ^−^)]/[(*I* ^+^)+(*I* ^−^)] (Parsons *et al.*, 2013 ▶)
Absolute structure parameter: 0.037 (18)

### 

Data collection: *APEX2* (Bruker, 2011 ▶); cell refinement: *SAINT* (Bruker, 2011 ▶); data reduction: *SAINT*; program(s) used to solve structure: *SIR2002* (Burla *et al.*, 2005 ▶); program(s) used to refine structure: *SHELXL2013* (Sheldrick, 2008 ▶); molecular graphics: *ORTEP-3 for Windows* (Farrugia, 2012 ▶) and *DIAMOND* (Brandenburg & Berndt, 2001 ▶); software used to prepare material for publication: *WinGX* (Farrugia, 2012 ▶).

## Supplementary Material

Crystal structure: contains datablock(s) I. DOI: 10.1107/S160053681303417X/lh5676sup1.cif

Structure factors: contains datablock(s) I. DOI: 10.1107/S160053681303417X/lh5676Isup2.hkl

Additional supporting information:  crystallographic information; 3D view; checkCIF report

## Figures and Tables

**Table 1:** Hydrogen-bond geometry (Å, °)

*D*—H⋯*A*	*D*—H	H⋯*A*	*D*⋯*A*	*D*—H⋯*A*
N1—H1*A*⋯Cl1^i^	0.99	2.50	3.327 (5)	141
N2—H1*N*⋯O2^ii^	0.86 (5)	2.10 (6)	2.891 (8)	152 (5)
N2—H2*N*⋯O1^iii^	0.86 (5)	2.18 (6)	3.015 (8)	163 (5)

Symmetry codes: (i) 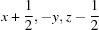; (ii) 
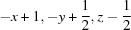
; (iii) 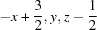.

**Fig. 1.**
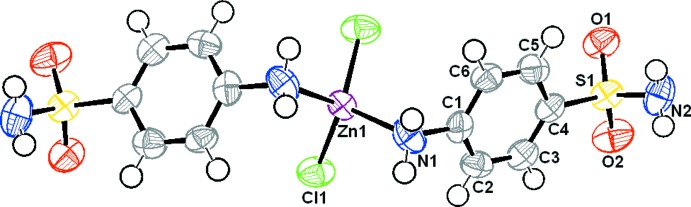
The molecular structure of (I), with displacement ellipsoids drawn at the 50% probability level. Only the the asymmetric unit is labelled. H atoms are represented as small spheres of arbitrary radii.

**Fig. 2.**
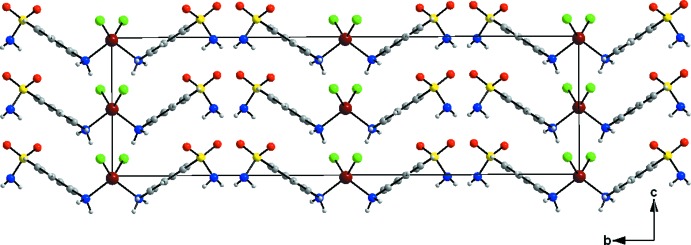
Packing of (I) viwed via a axis showing alterning layers in zigzag parallel to (001) planes.

**Fig. 3.**
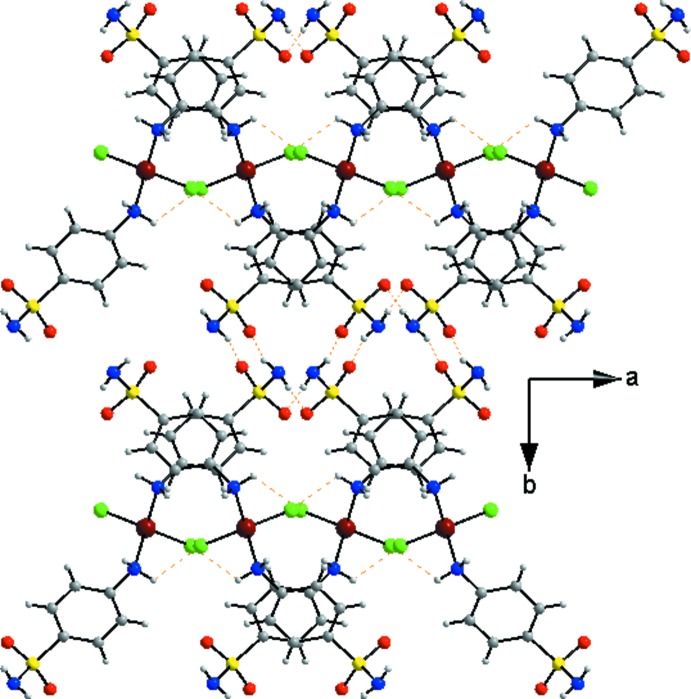
Packing of the title compound viewed along the c axis showing hydrogen bonds [N—H···Cl and N—H···O] as dashed lines

## supplementary crystallographic information

### 1. Comment

The use of metal complexes as pharmaceuticals has shown promise in recent years particularly as anticancer agents (Wong & Giandomenico, 1999). Recently, sulfadrugs and their complexes have applications as diuretic, antiglaucoma or antiepileptic drugs among others (Ferrer *et al.*, 1990; Supuran *et al.*, 1998). Furthermore, metal sulfanilamides have received attention owing to their antimicrobial activity (Medina *et al.*, 1999). As part of our ongoing studies on the synthesis, structures and biological activity of organometallic sulfanilamide complexes (Benmebarek *et al.* 2012, 2013) we have synthesized and determined the crystal structure of the title compound (I).

The molecular geometry and the atom-numbering scheme are shown in Fig 1. The title compound is a mononuclear zinc(II) complex in which the Zn^II^ ion is in a slightly distorted tetrahedral geometry involving two Cl atoms and two N atoms from the two amino groups of the sulfanilamide unit [Zn—Cl = 2.2288 (16) Å and Zn—N = 2.060 (5) Å]. The angles involving the Zn atom range from 104.38 (14) to 116.38 (11)°. The dihedral angle between the two benzene rings is 67.1 (3)°. The crystal packing can be describe by a interacting layers in zigzag parallel to (001) planes (Fig.2). The sulfonamidic nitrogen atoms, acting as donor, participate in intermolecular N—H···O and N—H···Cl hydrogen bonds formimg a three-dimensional network (Table 1, Fig. 3).

### 2. Experimental

ZnCl_2_ (0.1 mmol) and sulfanilamide (0.5 mmol) were dissolved in 15 ml solution of NaOH 0.01 N and heated, under continuous stirring, at 373 K for 15 min. The solution was transfered into a 23 ml teflon-lined stainless steel autoclave and heated at 453 K for 3 days. Then the autoclave was cooled to room temperature at 10K/h. Clear block-shaped crystal were collected, washed with ethanol and dried in air at ambient temperature.

### 3. Refinement

All non-H atoms were refined with anisotropic atomic displacement parameters. Approximate positions for all H atoms were first obtained from the difference electron density map. However, the H atoms were situated into idealized positions and the H-atoms have been refined within the riding atom approximation. The applied constraints were as follows: C—H = 0.93 Å and N—H = 0.90 Å. *U*_iso_ = 1.2*U*_eq_(C or N). The atoms H1N and H2N amino group were located in difference Fourier maps and included in the subsequent refinement with the constaint of N—H = 0.85 (2)Å and *U*_iso_(H) = 1.5U_eq_(N).

### Crystal data

**Table:**

[ZnCl_2_(C_6_H_8_N_2_O_2_S)_2_]	*D*_x_ = 1.774 Mg m^−^^3^
*M**_r_* = 480.68	Mo *K*α radiation, λ = 0.71073 Å
Orthorhombic, *A**b**a*2	Cell parameters from 805 reflections
*a* = 7.7957 (15) Å	θ = 2.9–22.3°
*b* = 27.916 (6) Å	µ = 1.92 mm^−^^1^
*c* = 8.2701 (17) Å	*T* = 150 K
*V* = 1799.8 (6) Å^3^	Block, colourless
*Z* = 4	0.23 × 0.19 × 0.15 mm
*F*(000) = 976	

### Data collection

**Table:**

Bruker APEXII CCD diffractometer	1563 reflections with *I* > 2σ(*I*)
Radiation source: sealed tube	*R*_int_ = 0.075
φ and ω scans	θ_max_ = 27.4°, θ_min_ = 2.9°
Absorption correction: multi-scan (*SADABS*; Bruker, 2002)	*h* = −10→10
*T*_min_ = 0.639, *T*_max_ = 0.746	*k* = −36→35
6151 measured reflections	*l* = −10→10
2051 independent reflections	

### Refinement

**Table:**

Refinement on *F*^2^	Hydrogen site location: mixed
Least-squares matrix: full	H atoms treated by a mixture of independent and constrained refinement
*R*[*F*^2^ > 2σ(*F*^2^)] = 0.040	*w* = 1/[σ^2^(*F*_o_^2^)] where *P* = (*F*_o_^2^ + 2*F*_c_^2^)/3
*wR*(*F*^2^) = 0.067	(Δ/σ)_max_ < 0.001
*S* = 1.00	Δρ_max_ = 0.38 e Å^−^^3^
2051 reflections	Δρ_min_ = −0.40 e Å^−^^3^
120 parameters	Absolute structure: Flack parameter determined using 601 quotients [(*I*^+^)-(*I*^-^)]/[(*I*^+^)+(*I*^-^)] (Parsons *et al.*, 2013)
3 restraints	Absolute structure parameter: 0.037 (18)

### Special details

**Table:**

Geometry. All esds (except the esd in the dihedral angle between two l.s. planes) are estimated using the full covariance matrix. The cell esds are taken into account individually in the estimation of esds in distances, angles and torsion angles; correlations between esds in cell parameters are only used when they are defined by crystal symmetry. An approximate (isotropic) treatment of cell esds is used for estimating esds involving l.s. planes.

### Fractional atomic coordinates and isotropic or equivalent isotropic displacement parameters (Å^2^)

**Table:**

	*x*	*y*	*z*	*U*_iso_*/*U*_eq_	
C1	0.1797 (7)	0.0904 (2)	−0.0925 (8)	0.0181 (15)	
C2	0.1224 (6)	0.1288 (2)	−0.0041 (9)	0.0202 (13)	
H2	0.0028	0.1339	0.0093	0.024*	
C3	0.2387 (8)	0.1598 (2)	0.0653 (8)	0.0205 (15)	
H3	0.2002	0.1866	0.1263	0.025*	
C4	0.4132 (7)	0.1514 (2)	0.0446 (8)	0.0182 (14)	
C5	0.4705 (7)	0.1121 (2)	−0.0415 (9)	0.0214 (16)	
H5	0.5901	0.1065	−0.0532	0.026*	
C6	0.3546 (7)	0.0811 (2)	−0.1103 (7)	0.0188 (15)	
H6	0.3928	0.0539	−0.1689	0.023*	
N1	0.0594 (5)	0.05705 (19)	−0.1656 (7)	0.0221 (13)	
H1A	0.1090	0.0447	−0.2676	0.026*	
H1B	−0.0475	0.0744	−0.1929	0.026*	
N2	0.6629 (6)	0.21766 (18)	−0.0154 (9)	0.0267 (12)	
H1N	0.591 (6)	0.235 (2)	−0.069 (7)	0.032*	
H2N	0.712 (7)	0.1973 (18)	−0.079 (7)	0.032*	
O1	0.6944 (5)	0.15998 (16)	0.2073 (6)	0.0275 (12)	
O2	0.4808 (6)	0.22535 (16)	0.2260 (6)	0.0312 (12)	
S1	0.56714 (18)	0.18997 (6)	0.1307 (2)	0.0204 (3)	
Cl1	−0.22591 (17)	0.02497 (6)	0.1242 (2)	0.0273 (4)	
Zn1	0.0000	0.0000	−0.01787 (14)	0.0176 (2)	

### Atomic displacement parameters (Å^2^)

**Table:**

	*U*^11^	*U*^22^	*U*^33^	*U*^12^	*U*^13^	*U*^23^
C1	0.023 (3)	0.019 (4)	0.012 (3)	−0.003 (3)	−0.004 (3)	0.007 (3)
C2	0.020 (3)	0.024 (3)	0.017 (4)	0.004 (2)	0.002 (3)	0.005 (4)
C3	0.027 (3)	0.020 (4)	0.015 (3)	0.001 (3)	−0.001 (3)	0.002 (3)
C4	0.025 (3)	0.016 (3)	0.014 (3)	0.001 (3)	−0.002 (3)	0.001 (3)
C5	0.021 (3)	0.024 (3)	0.019 (4)	0.002 (3)	0.005 (3)	0.004 (3)
C6	0.022 (3)	0.018 (4)	0.017 (4)	0.004 (3)	0.005 (3)	−0.002 (3)
N1	0.018 (2)	0.031 (3)	0.017 (3)	−0.004 (2)	−0.001 (2)	0.001 (3)
N2	0.039 (3)	0.023 (3)	0.019 (3)	−0.007 (2)	−0.002 (4)	0.003 (4)
O1	0.030 (3)	0.025 (3)	0.027 (3)	0.003 (2)	−0.011 (2)	0.000 (2)
O2	0.041 (3)	0.027 (3)	0.026 (3)	0.007 (2)	−0.006 (2)	−0.012 (2)
S1	0.0261 (7)	0.0209 (9)	0.0144 (8)	0.0013 (6)	−0.0036 (8)	−0.0010 (9)
Cl1	0.0259 (7)	0.0307 (9)	0.0252 (9)	0.0073 (7)	0.0086 (9)	0.0030 (10)
Zn1	0.0164 (4)	0.0218 (5)	0.0145 (5)	−0.0010 (5)	0.000	0.000

### Geometric parameters (Å, º)

**Table:**

C1—C2	1.371 (8)	N1—Zn1	2.060 (5)
C1—C6	1.396 (7)	N1—H1A	0.9900
C1—N1	1.454 (7)	N1—H1B	0.9900
C2—C3	1.380 (8)	N2—S1	1.617 (6)
C2—H2	0.9500	N2—H1N	0.86 (3)
C3—C4	1.391 (8)	N2—H2N	0.86 (3)
C3—H3	0.9500	O1—S1	1.444 (4)
C4—C5	1.382 (8)	O2—S1	1.432 (5)
C4—S1	1.763 (6)	Cl1—Zn1	2.2288 (16)
C5—C6	1.374 (8)	Zn1—N1^i^	2.060 (5)
C5—H5	0.9500	Zn1—Cl1^i^	2.2288 (16)
C6—H6	0.9500		
			
C2—C1—C6	121.3 (6)	Zn1—N1—H1A	108.9
C2—C1—N1	120.8 (5)	C1—N1—H1B	108.9
C6—C1—N1	117.9 (6)	Zn1—N1—H1B	108.9
C1—C2—C3	119.9 (5)	H1A—N1—H1B	107.8
C1—C2—H2	120.1	S1—N2—H1N	110 (4)
C3—C2—H2	120.1	S1—N2—H2N	110 (4)
C2—C3—C4	119.0 (6)	H1N—N2—H2N	110 (7)
C2—C3—H3	120.5	O2—S1—O1	118.8 (3)
C4—C3—H3	120.5	O2—S1—N2	107.4 (3)
C5—C4—C3	120.9 (6)	O1—S1—N2	106.7 (3)
C5—C4—S1	118.2 (4)	O2—S1—C4	108.9 (3)
C3—C4—S1	120.8 (5)	O1—S1—C4	106.9 (3)
C6—C5—C4	120.0 (5)	N2—S1—C4	107.7 (3)
C6—C5—H5	120.0	N1^i^—Zn1—N1	107.2 (3)
C4—C5—H5	120.0	N1^i^—Zn1—Cl1^i^	104.38 (14)
C5—C6—C1	118.8 (6)	N1—Zn1—Cl1^i^	112.14 (13)
C5—C6—H6	120.6	N1^i^—Zn1—Cl1	112.14 (13)
C1—C6—H6	120.6	N1—Zn1—Cl1	104.38 (14)
C1—N1—Zn1	113.2 (4)	Cl1^i^—Zn1—Cl1	116.38 (11)
C1—N1—H1A	108.9		
			
C6—C1—C2—C3	1.9 (10)	N1—C1—C6—C5	−179.7 (6)
N1—C1—C2—C3	179.5 (6)	C2—C1—N1—Zn1	−91.0 (6)
C1—C2—C3—C4	−0.3 (10)	C6—C1—N1—Zn1	86.8 (6)
C2—C3—C4—C5	−1.2 (10)	C5—C4—S1—O2	−174.7 (5)
C2—C3—C4—S1	−180.0 (5)	C3—C4—S1—O2	4.1 (6)
C3—C4—C5—C6	1.1 (10)	C5—C4—S1—O1	−45.2 (6)
S1—C4—C5—C6	179.9 (5)	C3—C4—S1—O1	133.6 (5)
C4—C5—C6—C1	0.5 (10)	C5—C4—S1—N2	69.1 (6)
C2—C1—C6—C5	−2.0 (10)	C3—C4—S1—N2	−112.0 (5)

Symmetry code: (i) −*x*, −*y*, *z*.

### Hydrogen-bond geometry (Å, º)

**Table:**

*D*—H···*A*	*D*—H	H···*A*	*D*···*A*	*D*—H···*A*
N1—H1*A*···Cl1^ii^	0.99	2.50	3.327 (5)	141
N2—H1*N*···O2^iii^	0.86 (5)	2.10 (6)	2.891 (8)	152 (5)
N2—H2*N*···O1^iv^	0.86 (5)	2.18 (6)	3.015 (8)	163 (5)

Symmetry codes: (ii) *x*+1/2, −*y*, *z*−1/2; (iii) −*x*+1, −*y*+1/2, *z*−1/2; (iv) −*x*+3/2, *y*, *z*−1/2.
